# Relative Density of Away from Home Food Establishments and Food Spend for 24,047 Households in England: A Cross-Sectional Study

**DOI:** 10.3390/ijerph15122821

**Published:** 2018-12-11

**Authors:** Tarra L. Penney, Thomas Burgoine, Pablo Monsivais

**Affiliations:** 1 UKCRC Centre for Diet and Activity Research (CEDAR), MRC Epidemiology Unit, University of Cambridge School of Clinical Medicine, Box 285 Institute of Metabolic Science, Cambridge Biomedical Campus, Cambridge CB2 0QQ, UK; tb464@medschl.cam.ac.uk; 2 Department of Nutrition and Exercise Physiology, Elson S. Floyd College of Medicine, Washington State University, Spokane, WA 99164, USA; p.monsivais@wsu.edu

**Keywords:** food availability, eating away from home, household food spending

## Abstract

Eating away from home is a risk factor for poor diet quality and obesity. With an ever-increasing proportion of household food spend directed toward eating out, the proliferation of these food establishments may contribute to their use, a potential precursor to less healthy food choices and low overall diet quality. However few studies are conducted at the national level and across a range of away from home food sources. The purpose of this study was to examine the association between the density of away from home food establishments (e.g., restaurants, fast food outlets and cafés) and household spend on away from home food within a nationally representative sample for England, UK. A cross-sectional analysis of data from Wave 1 of the UK Household Longitudinal Survey (*n* = 24,047 adults aged ≥19y) was conducted. Exposure was characterised as the density of away from home food establishments to all other food sources within 1 mile of the home, divided into quintiles (Q1 as lowest exposure and Q5 as highest exposure). The primary outcome included households with a high away from home equivalised monthly food spend (≥25% of total food spend). Logistic regression was used to estimate associations between away from home food establishment exposure and high away from home food spend. Away from home food establishment density was significantly associated with a greater odds of high monthly food spend (Q3: OR = 1.18, 95% CI = 1.07, 1.30; Q4: OR = 1.30, 95% CI = 1.18, 1.43; and Q5: OR = 1.52, 95% CI = 1.37, 1.68) with attenuation after controlling for known socioeconomic confounders (Q4: OR = 1.13, 95% CI = 1.02, 1.25; and Q5: OR = 1.16, 95% CI = 1.04, 1.30) compared to those least exposed (Q1). Those most exposed to away from home food establishments had a 16% greater odds of allocating more than 25% of household food spend on away from home food sources. This study provides one of the first analyses at the national level to examine the role of the local food environment in relation to household food spend, a potential precursor to diet quality and health.

## 1. Background

Unhealthy diet and obesity are considered global epidemics that present a significant challenge for public health and policy action [1]. The 2013 global burden of disease study ranked unhealthy diet and high body weight as the first and third contributors, respectively, to morbidity and mortality [2]. Unhealthy food and nutrient intake include a low intake of fruits and vegetables, whole grains, low fat milk, fibre, nuts and seeds and high intake of red and processed meat, sugar and sodium [3]. Consumption of unhealthy foods, nutrients and poor overall diet quality are often observed among those who eat frequently at retail food establishments such as sit down restaurants, fast food outlets and coffee shops or cafés [4,5,6]. Away from home food sources can be appealing as they tend to serve large energy-dense portions, creating a low calorie-to-cost ratio [7] that can contribute to a greater risk of obesity, higher body weight and weight gain over time among frequent users [8,9].

Recently public health efforts to reduce the impact of eating away from home on population level diet quality and risk of obesity have focused on addressing retail ‘food swamps’, a concept characterised by a disproportionally high density of unhealthy to healthy food sources within a geographic area [10]. While there is significant debate regarding what food outlets are healthy or unhealthy, proliferation of a range of retail food establishments themselves (i.e., the sheer density) is suggested to influence food choice independent of individual level factors via easy and convenient access, thereby making them more likely to be chosen as a food source [11]. The dual process model of food choice supports this view suggesting that choice is the result of both an intentional process that favours convenience, preferences, tastes [12] and sensitivity to price [11] and an automatic process, where a flood of sensory cues can encourage people to eat even when they may not require food [13].

**Figure 1:**
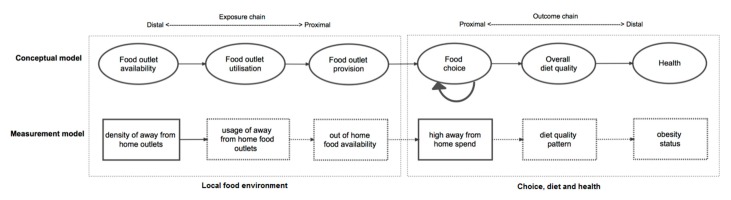
Conceptual model describing how the local food environment might relate to diet and health, highlighting measurement model for study hypothesis.

Empirical studies examining the local food environment and health often include the implicit assumption that a direct and independent effect on behaviour exists. However the influence of the density of away from home food establishments on food choice might be more accurately theorised as one distal environmental exposure on a causal chain that includes a range of intermediate exposures and outcomes. In combination these factors along the exposure chain provide the structural conditions needed for the frequent utilisation of away from home food establishments, followed by exposure to the food provision within outlets and subsequent food choice (see Figure 1) [14]. It is then through repeating these food choices over time that a dietary pattern emerges that can ultimately influence health. 

The evidence for the role of the local food environment on food choice and diet quality is plagued by conceptual and methodological challenges. The number of food outlets providing food for consumption away from home has been increasing [15] along with a rise in household spend on away from home sources [16]. However much of the research that examines the link between environmental exposures with diet and health remains equivocal. A proliferation of fast food outlets in one’s neighbourhood has been shown to be associated with take away food intake and obesity [17,18,19,20]. One study from the US showed that for every standard deviation increase in fast-food exposure, the odds of consuming fast food near home increased 11%–61%, and the odds of a healthy diet decreased 3–17% (depending on adjustment) [21]. However, other studies from Canada [22] and the US have shown no association between density and proximity of exposure to fast food outlets and fast food purchasing after adjustment [23]. In addition to the mixed nature of the evidence base, several studies have been restricted to regional geographies [19,20], with a need to examine these relationships in nationally-representative samples [18,19,20]. 

The purpose of this cross-sectional study was to examine the association between exposure to away from home food outlets (e.g., restaurants, fast food outlets and cafés) and food spend on away from home sources within a nationally-representative sample for England. 

## 2. Methods

### 2.1. Data Source and Participants

Data from the UK Household Longitudinal Study (UKHLS) were used [24]. UKHLS is a nationally-representative longitudinal panel survey of UK adults which began in 2009 and included over 40,000 households (57% household and 82% individual response rates) [25]. Details on the study and its sampling strategy are reported elsewhere [26]. In short, participants are surveyed annually to collect demographic, socioeconomic, behaviour and health related information using a computer assisted personal interview. The household questionnaire is answered by a reference person (see Supplementary Materials for sampling strategy, Figure S1) [25]. 

For this analysis the general population sample was restricted to English households with data reported by an adult (aged 19 years or older) reference person (*n* = 24,711) with complete household food spend for home and away from home sources (*n* = 24,047) (see Supplementary Materials for sample flow diagram, Figure S2.). The full household and analytic sample did not differ significantly on key demographic, socioeconomic, exposure or outcome variables; therefore only the analytic sample is presented. Ethical approval was not required for the analysis of secondary data presented here, but was obtained by UKHLS for data collection.

### 2.2. Exposure: Density of Away from Home Food Outlets to All Food Outlets

Data on the location of food establishments were obtained from Ordnance Survey’s Points of Interest (POI), an administrative dataset for use by government and business [27]. The data are created and maintained by PointX, which sources the data from a list of over 150 suppliers, runs verification checks and classifies the features (see the reference for user guide) [28]. POI data has been found to be a viable alternative to accurate local council data in the UK [29]. Each food outlet is provided with geographic coordinates at a stated accuracy of within 1 m. The data is updated quarterly; this analysis used data from June 2014. The use of POI data for determining food environment exposure has been demonstrated in previous studies [30,31,32]. Away from home food establishments were comprised of three subcategories: ‘sit-down restaurants’, ‘fast food outlets’ and ‘cafés’. Example sit-down restaurants include Bella Italia, Wetherspoons or Nando’s; fast food outlets include McDonald’s, Burger King or Kentucky Fried Chicken; and cafés include Café Nero, Starbucks and Costa. Food outlets primarily used as a food source for at home food preparation (e.g., supermarkets, convenience and green grocers etc.) were classified as ‘Other’ (see Supplementary Materials for food outlet frequency and classification, Table S1). Retail outlets in which food provision is not the primary service or food is not sold directly to the public were excluded (e.g., workplace cafeterias, cinemas and recreation facilities). 

**Figure 2:**
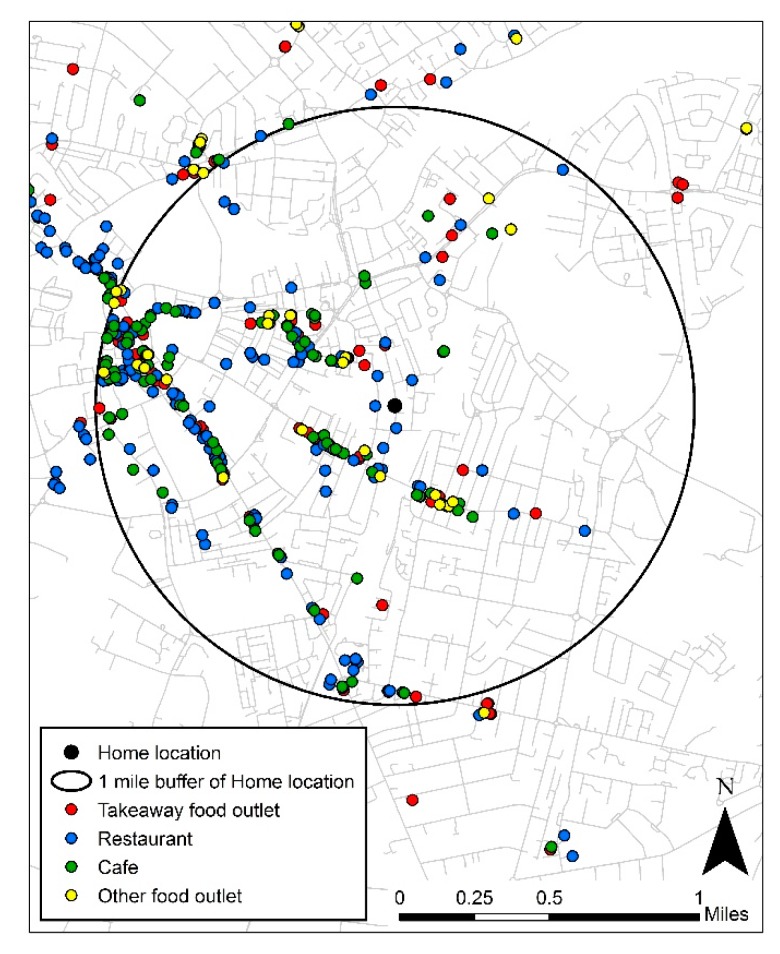
Depiction of how exposure was characterised as proportion of away from home food outlets to all food outlets around the home.

Using a geographic information system (ArcGIS 10, ESRI), relative away from home food establishment density was calculated for each household. Relative density is theorised as a spatial metric representing the intensity of exposure to features of the local food environment [33], and consistent with the focus on examining the role of density of away from home food establishments for this analysis [10]. Food outlet counts (away from home and other food outlet types) were made within a 1 mile Euclidean (straight line) radius buffer, centred on household addresses provided through UKHLS secure data access (see Figure 2). This distance is based upon previous work suggesting a behavioural relevance to food shopping among UK adults [34]. Relative density for each household was then calculated as the sum of the count of away from home establishments divided by the count of all food sources, divided into quintiles. Q1 represented those with the lowest proportion of away from home food outlets and Q5 representing those with the highest proportion. 

### 2.3. Outcome: Household with High Away from Home Food Spend

Household food expenditure was self-reported using two questions in the UKHLS survey: “About how much has your household spent in total on food and groceries in the last four weeks from a supermarket or other food shop or market? Please do not include alcohol” and “About how much have you and other members of your household spent in total on meals or snacks purchased outside the home in the last four weeks?” Monthly household food spend (£) was equivalised against household size using the OECD modified equivalence scale [35] and top-coded at the limit of the second highest decile (£500/month) that is, all values ≥£500 were recoded at that value. Sensitivity analyses informed the top coding through comparing mean home and away from home food spend to household spend estimates from the UK’s Living Costs and Food survey for 2009 [36]. Total away-from-home food spend was divided by total household food spend then stratified into tertiles. The highest tertile (T3) was used to define households with a ‘high’ proportion of their total monthly household food spend on away from home food (approximately 25% or more). While no validation study of UKHLS data has been performed, previous work has found no significant differences between receipts and self-reported data on total food expenditures, expenditures at food stores, or eating out [37].

### 2.4. Additional Covariates

Demographic factors including age, categorised as young adult (18–35 years), middle-age (36–50 years) and older-age (>50 years), and sex of the household reference person were self-reported. Additionally, two indicators of socioeconomic status were patterned by both exposure and outcome: educational attainment categorised as ‘None, GCSE or equivalent (≤11 years)’, ‘A-level (12–13 years)’, ‘Vocational qualifications (12–13 years)’, ‘Degree or higher (>13 years)’ or ‘Missing’, and equivalised household income categorised as ‘£14,999 or below’, ‘£15,000–£24,999’, ‘£25,000–£34,999’, ’£35,000–£49,999’, ‘£50,000 and above’ or ‘Missing.’ Missing covariates were examined across all exposure variables, with no significant differences in percentages across exposure levels found. As a result, we included cases with missing covariate data in appropriate models to improve estimates and avoid case deletion (i.e., the missing indicator approach) [38]. 

### 2.5. Statistical Analyses

Descriptive statistics were used to summarise demographic, socioeconomic and household food spend variables, both overall and across away from home food outlet exposure levels. Study weights for Wave 1 cross-sectional analyses, prepared and provided by UKHLS, were used to account for participant non-response and clustered study design. Therefore, weighted mean percentages (with 95% CIs) are presented rather than raw frequencies.

Binary logistic regression was used to model high away from home food spend households by quintile of away from home food outlet density (Model 0). Crude models were adjusted for demographic variables (age and sex), proportion of restaurants, proportion of fast food outlets and proportion of cafés, total number of other food sources and total equivalised household food spend (Model 1). Lastly, Model 1 was additionally adjusted for socioeconomic variables (education and income) (Model 2). All statistical analyses were carried out in Stata version 14 (StataCorp LP., College Station, TX, USA) [39].

### 2.6. Sensitivity Analyses

Alternate multivariate models were used to examine sensitivity of our results to model specification. Additional covariate specifications were examined including with education as single indicator of socioeconomic status, ethnicity (White, all others) and rurality (Urban or Rural), and the removal of proportions of restaurants, fast food outlets and cafés. 

## 3. Results

The weighted sample characteristics presented in Table 1 indicate that half the sample were female (50.6%), with representation across socioeconomic groups (30.8% having ‘No or compulsory’ education and 23.3% with ‘Higher education’). Additionally, 40.7% of the sample had an equivalised income less than £14,999, with 5.7% having an income above £50,000. On average, away from home food outlet density was nearly 60% (of all outlets), composed of a mixture of 28.8% restaurants, 22.6% fast food outlets and 8.3% cafés. Average total food spend was £204 per month, with 17.6% of that spend being directed toward away from home food sources. 

Table 2 shows selected weighted sample demographic and socioeconomic variables across level of away from home food outlet density. For the highest level of exposure to away from home food outlet density, a higher proportion of the sample are younger, belonged to a more socioeconomically-advantaged group (higher educational attainment and income), had higher total food related spend and allocated a greater proportion of household food spend toward away from home sources. Sex and mean age showed similar proportions of the sample across exposure levels. 

**Figure 3:**
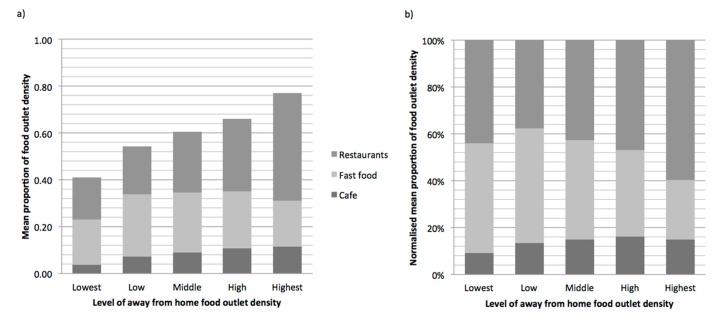
(**a**) Stacked weighted mean % of food outlet density by quintile of away from home exposure for each food outlet type; (**b**) Normalised contribution of weighted mean % food outlet density for type of food outlet by proportion of away from home exposure quintile.

Across quintile of away from home food outlet exposure, absolute density of food outlets range from just over 40% to more than 76% of total food outlet exposure (Figure 3a). The relative contribution of the three away from home food outlet types varied across quintiles, with an overall higher contribution of restaurants from lowest to highest quintile of exposure, where that outlet type provides the dominant source of away from home outlet exposure in the highest quintile (Figure 3b).

Regression analyses (Table 3) showed that compared to the lowest level of away from home food outlet density (Q1) higher levels of density were associated with a greater odds of high monthly away from home food spend (Q3: OR = 1.18, 95% CI = 1.07, 1.30; Q4: OR = 1.30, 95% CI = 1.18, 1.43; and Q5: OR = 1.52, 95% CI = 1.37, 1.68) (Model 0). 

This relationship was attenuated after adjustment for known confounders with Q3 becoming non-significant (Q3: OR = 1.09, 95% CI = 0.99, 1.21) but with Q4 and Q5 remaining significantly associated with a greater odds of high away from home food spend (Q4: OR = 1.13, 95% CI = 1.02, 1.25 and Q5: OR = 1.16, 95% CI = 1.04, 1.30, respectively) (Model 2).

Sensitivity analyses with alternative model specifications were performed as described but results did not differ meaningfully from those described here. 

## 4. Discussion

Using a nationally-representative sample of UK households, the purpose of this work was to contribute to our understanding to whether exposure to away from home food establishments in residential neighbourhoods is a precursor to population-level food choice. We observed that exposure to the greatest density of away from home food establishments, relative to all food sources, was associated with greater odds of households directing a high proportion of their monthly food spend on away from home food. Specifically, after adjustment, those in the most-exposed group had 16% higher odds of being high-spending households with respect to away from home food sources. 

Our findings, although based on economic outcomes, provide evidence of an important but neglected theoretical link between the local food environment and health. Previous studies have observed associations between residentially-based estimates of fast food access and dietary and/or weight outcomes [18,19,20]. This work demonstrates an association with a more proximal outcome of food spending in particular and across a wider range of away from home food establishments. While our study had no direct measures of the health-related characteristics of food spend, other research has found that eating out-of-home is associated with lower diet quality and poor health outcomes [5,40]. 

The demonstration of a consistent relationship between the local food environment, diet and health remains somewhat elusive [41,42,43]. Previous research has suggested much of this heterogeneity may be due to methodological differences in exposure estimation, including the various decisions made surrounding the selection of spatial measures, geographic units, buffers around individual addresses [41,42] and the quality of dietary outcome measurement [42,43]. While the findings here should be interpreted within the context of a mixed evidence base that is yet unexplained, the findings do support previous work that has reported a significant relationship between exposure to away from home food establishments, diet and weight, and the amplification of inequalities [19,20,44]. However, a degree of caution may be warranted when using the observed association found here to *infer how a change* in the local food environment might influence diet and health. There is a paucity of studies available that examine the impact of a change in away from home food outlet exposure on diet or health, or address issues that could undermine causal inference including the role of neighbourhood self-selection (i.e., individual preferences drive selection of a neighbourhood that provides a local food environment that supports their preferences) [45]. 

In addition to structural factors, personal food tastes and preferences might play an important role in understanding differences in the relationship between exposure to away from home food sources and diet [44]. For example, consumers report preferences for fast food outlets that are convenient, easy to access and provide tasty foods, with availability of nutritious foods being the least important factor [46]. Additionally, individual preferences may be socioeconomically patterned, with more advantaged individuals possessing the material, psychosocial and time related resources needed to select food outlets and food items of their choosing regardless of what is easily accessible [47,48]. In our findings, higher income and more-educated individuals tended to have higher away from home food spend, suggesting a preference for the service and convenience of restaurants, cafes and fast food outlets among these population groups. 

### 4.1. Policy Implications

As discussed, these research findings contribute to a growing scientific evidence base, which suggests that greater access to away from home food establishments contributes to unhealthy dietary behaviour, excess body weight and obesity [20,49,50] Although some questions remain, this growing literature has direct links to public health policy through informing ‘healthy’ neighbourhood design [51], which is increasingly understood by planners as a low-agency, population-level public health intervention [52]. For example, planners in English local government are actively encouraged to implement planning laws that limit growth in the fast food sector [53], focussing on areas of perceived need, where levels of obesity are currently high, or where existing access to fast food outlets is sufficient. Internationally, there are examples of similar practice [54] and anecdotal evidence to suggest that implementation of such policies is increasingly commonplace [55]. The effectiveness of many of these policies is yet to be determined. 

### 4.2. Methodological Considerations and Limitations

For this study, food outlet exposure was estimated within residential neighbourhoods, defined as within 1 mile of each participant’s home address. This home-based characterisation may overestimate some forms of outlet exposure, particularly if there are physical barriers in the environment not accounted for using the buffer method, and underestimate other forms of exposure, particularly for food outlets accessed from the workplace or while commuting between locations [20]. Additionally, the OS POI data on food outlet locations is not a routinely validated data set. Approximately 60% of the data is reported as ‘ground truthed’, however given the size of the data, it is not feasible to check each location. We used 2014 data with the assumption that secular food outlet change is relatively slow, and therefore unlikely to result in a significant difference in quintile of exposure between the year the food outlet data was collected and the year the survey data was collected, however this assumption is not based on a validation study for the UK. Additionally, the use of self-report data for estimates of household food spending is an important consideration. Although validation of self-report expenditure has been done in previous work [37], there is no UKLHS validation study for household food expenditure that we are aware of. Household food spend was benchmarked against Living Cost and Food data, however, while these adjusted data are likely appropriate for the type of analysis presented here, they may not provide perfectly accurate population level estimates of household food spend. Also, while we have adjusted for known confounders that were available to us and used routinely in previous research, the role of other unmeasured confounders cannot be ruled out, including car ownership or access to or use of public transportation. 

Major strengths of this study include the use of a nationally-representative geographically diverse sample of UK households, the use of objectively measured food outlet data and coherence between the characterisation of the food environment and outcome. Using data from this national survey increases the generalizability of our findings to the UK population. Previous studies have typically used data from geographically-circumscribed samples from study cohorts. Although such samples can be representative of their underlying regional populations, they may be more limited in terms of generalizability to a national context. For the first time in the published literature, we exploited novel household food spend data within this national social and economic panel survey, including information on the amount spent purchasing foods specifically for consumption away from home. 

## 5. Conclusions

Our findings suggest that those most exposed to away from home food establishments had a greater odds of allocating a high proportion of household food spend on away from home food sources. This study provides one of the first analyses at the national level to examine the role of the local food environment in relation to household food spend, a potential precursor to diet quality and health. Further research is required to better understand how and why different populations interact with their local food environment over time to inform the most effective policies to support healthy food choice while eating away from the home, diet and health. 

## Figures and Tables

**Table 1:** Weighted sample characteristics for analytical sample (*n* = 24,047) from Wave 1 as percentage or mean and 95% CI where indicated.

	N (Unweighted)	Men	Women	All
		10,236	13,811	24,047
Demographic				
	Age in years ^a^	50.5 (50.2–50.8)	50.6 (50.2–51.0)	50.3 (50.0–50.7)
	18–35 years	22.2	23.5	22.9
	36–50 years	29.7	30.8	30.3
	Over 50 years	48.1	45.7	46.8
Socioeconomic				
Education	None	14.7	20.5	17.9
	GCSE or equivalent (≤11 years)	28.2	32.88	30.8
	A-level (12–13 years)	19.2	13.7	16.2
	Vocational qualifications (12–13 years)	10.3	12.8	11.7
	Degree (>13 years)	27.4	20.0	23.3
	Missing	0.40	0.28	0.35
Equivalised household Income	<£14,999	44.4	36.2	40.7
	£15,000–£24,999	28.2	28.9	29.1
	£25,000–£34,999	13.9	16.3	15.0
	£35,000–£49,999	7.81	10.9	9.26
	£50,000+	4.49	7.23	5.74
	Missing	0.17	0.27	0.22
Density of AFH food establishments				
	Restaurant to all other	29.1 (28.6–29.5)	28.5 (28.1–29.0)	28.8 (28.4–29.1)
	Fast food to all other	22.3 (22.0–22.7)	22.7 (22.4–23.1)	22.6 (22.3–22.9)
	Cafe to all other	8.39 (8.16–8.61)	8.14 (7.93–8.36)	8.25 (8.07–8.44)
	Total AFH establishments to all other	59.8 (59.4–60.3)	59.5 (59.1–60.0)	59.6 (59.3–60.0)
Household food Spend				
	Total Equivalised food spend/mo. ^a^	£209 (207–211)	£200 (198–202)	£204 (203–206)
	Percent AFH food spend/mo. ^a^	19.4 (19.1–19.8)	16.1 (15.8–16.4)	17.6 (17.4–17.9)

^a^ weighted mean (95% CI); AFH = Away from home.

**Table 2:** Weighted sample characteristics (*n* = 24,047) as column percentages (unless otherwise stated) by quintile of proportion of away from home food outlet density.

		Quintile of % Away from Home Food Outlet Exposure					
		Q1 = Lowest	Q2	Q3	Q4	Q5 = Highest	All
	N (unweighted)	5177	4785	4871	4697	4517	24,047
	Density of AFH establishments (min–max)	0–0.50	0.50–0.57	0.57–0.63	0.63–0.69	0.69–1	0–1
Demographic							
	Age in years ^a^	52.1 (51.4–52.7)	50.2 (49.5–50.9)	50.2 (49.6–50.9)	50.2 (49.5–50.9)	49.8 (49.0–50.5)	50.5 (50.2–50.8)
	18–35 years	19.8	22.5	22.9	24.7	24.9	22.9
	36–50 years	29.6	32.1	31.2	29.4	29.4	30.3
	Over 50 years	50.7	45.5	45.9	45.9	45.7	46.8
	Sex (% Male)	44.1	45.2	44.5	46.3	47.3	45.5
Socioeconomic							
Education	None	19.4	19.6	19.6	17.0	14.1	17.9
	GCSE or equivalent (≤11 yrs)	33.8	31.8	31.2	30.9	25.9	30.8
	A-level (12–13 yrs)	16.2	16.8	16.2	15.7	16.4	16.2
	Vocational qualifications (12–13 yrs)	12.3	11.4	11.2	11.0	12.5	11.7
	Degree (>13 yrs)	18.1	20.3	21.8	25.3	31.1	23.3
Income	<£14,999	42.5	44.8	41.1	38.3	37.2	40.7
	£15,000–£24,999	30.2	28.5	29.7	29.3	27.6	29.1
	£25,000–£34,999	14.2	14.3	15.0	15.9	15.7	15.0
	£35,000–£49,999	8.28	8.30	8.80	10.2	10.6	9.26
	£50,000+	4.61	4.11	5.07	5.14	8.63	5.74
Household food spend							
	Total Equivalised food spend/mo. ^a^	£207 (198–204)	£196 (193–199)	£201 (198–204)	£206 (202–209)	£217 (213–221)	£204 (203–206)
	Percent AFH food spend/mo. ^a^	15.9 (15.4–16.5)	16.5 (15.9–17.7)	17.8 (17.2–18.4)	18.3 (17.7–18.9)	19.5 (18.8–20.1)	17.6 (17.4–17.8)

^a^ weighted mean (95% Confidence Interval); AFH = Away from home.

**Table 3:** Odds ratios and 95% confidence intervals for high % (top tertile) of away from home food spend (N = 24,047) by quintile of proportion of away from home food outlets exposure.

Proportion of Food Outlet Density	Odds of High Monthly % Away from Home Food Spend ^1^		
	Model 0	Model 1 ^2^	Model 2 ^3^
**Away from home**			
Q1	1.00 (-)	1.00 (-)	1.00 (-)
Q2	0.99 [0.90, 1.10]	0.97 [0.87, 1.07]	0.96 [0.86, 1.06]
Q3	1.18 *** [1.07, 1.30]	1.12 * [1.02, 1.24]	1.09 [0.99, 1.21]
Q4	1.30 *** [1.18, 1.43]	1.20 *** [1.08, 1.33]	1.13 * [1.02, 1.25]
Q5	1.52 *** [1.37, 1.68]	1.26 *** [1.13, 1.41]	1.16 ** [1.04, 1.30]

^1^ % of away from home food spend was divided into tertiles, with the highest tertile being ‘high’ in % of away from home food spending; ^2^ Adjusted for age, sex, total number of food outlets for restaurant, fast food, cafe, other and equivalised total food spend; ^3^ Additionally adjusted for education and equivalised income. * *p* < 0.05, ** *p* < 0.01, *** *p* < 0.001.

## Supplementary Materials

The following are available online at http://www.mdpi.com/1660-4601/15/12/2821/s1, Figure S1: Sample strategy for UKHLS, Figure S2: Analytic sample flow diagram, Figure S3: Food outlet classification and away from home food outlet groups for England, UK.
