# Supplementary material for: Relative Density of Away from Home Food Establishments and Food Spend for 24,047 Households in England: A Cross-Sectional Study
Source: Int J Environ Res Public Health. 2018 Dec 11;15(12):2821. doi: 10.3390/ijerph15122821 (PMC6313448; doi:10.3390/ijerph15122821)
Supplement: Supplementary file 1 [file ijerph-15-02821-s001.pdf]

Supplementary Figure S1. Sample strategy for UKHLS.

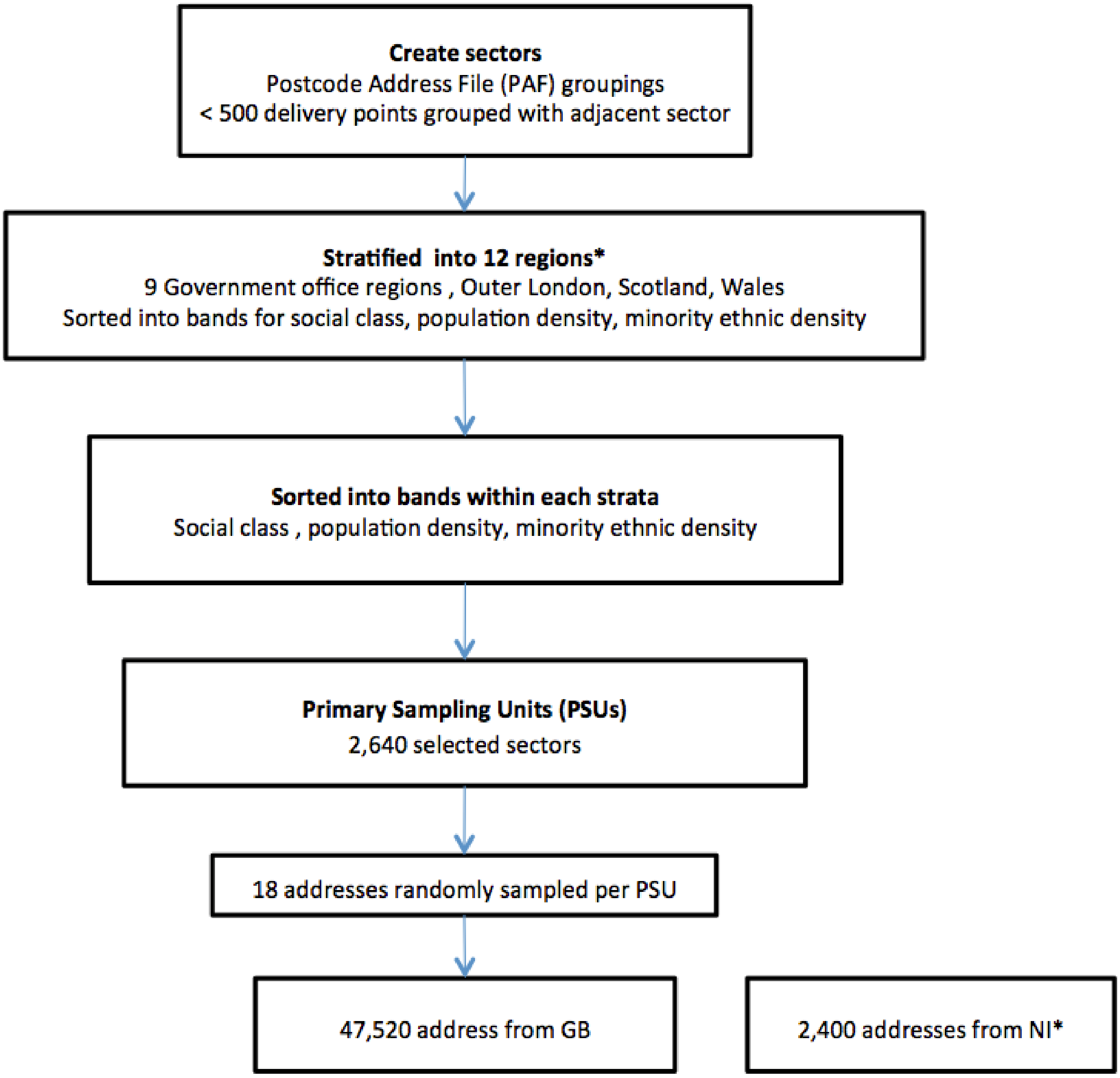

* NI was not included in stratified sampling

Supplementary Figure S2. Analytical Sample Flow Diagram

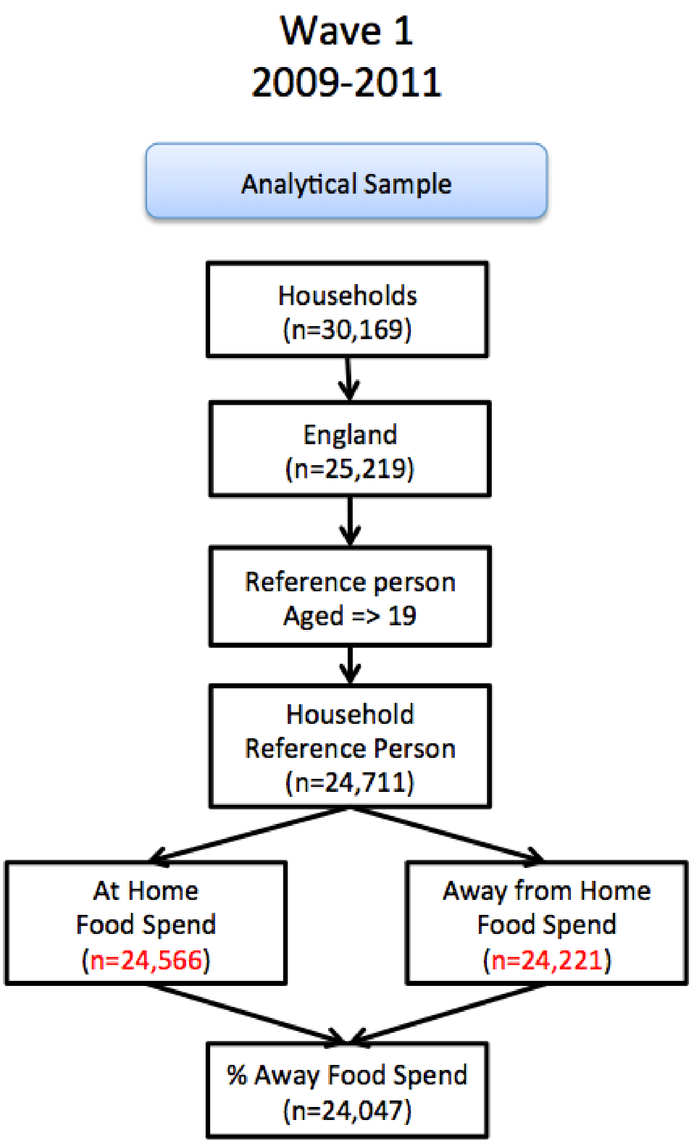

Supplementary Table 1. Distribution by OS Food outlet classification and away from home food outlet groups for England, UK.

POI data are provided with a range of variables, methods of measurement and sources including a unique reference number, feature name, classification code, latitude, longitude, positional accuracy rating and address verification.

**Food outlet type**		**Frequency**	**%**
Away from home			*64*
	Restaurants, Pubs and Bars	66,776	31
	Fast food, Takeaway, Fish & Chip	46,011	22
	Cafe and sandwich	23,135	11
All others			*36*
	Supermarket chains	10,541	5
	Convenience and Independent	36,110	17
	Baking and confection	11,039	5
	Special grocers	18,907	9
		**212,519**	**100**

Geo-FERN (Geographic Information System Food Environment ReportiNg) Checklist		
**INSTRUCTIONS**		
For each reporting item, insert a tick or cross in the shaded box to indicate whether the item has been reported, or insert 'N/A' if not applicable. Shading indicates whether items are essential or desirable. Reporting items can be included in supplementary materials if word limits are tight and if allowed by the publisher.		
**1. FOOD OUTLET DATA**	**Essential**	**Desirable**
Name of the data creator (e.g. 'Yellow Pages', 'Dunn & Bradstreet' etc.).	**X**	
Collection and/or publication year of the data (include both if known).	**X**	
Title of the dataset.	**X**	
Digital identifier of the dataset (e.g. a web address or DOI).		**X**
Publisher of the dataset.		**X**
Scope of the dataset (i.e. the geographic coverage of the dataset e.g. 'national' or 'regional' and the range of businesses included in the dataset, including any notable exclusions).		**X**
Identification of the data fields used in analyses.		**x**
Original purpose of the data (e.g. food hygiene regulation enforcement or commercial business data).		**X**
Methods used by the data creator to collect the data/compile the dataset (e.g. audits conducted by data creator).		**X**
Prevalence of missing data (e.g. number of entries with incomplete address information).		**N/A**
Methods for handling missing data (e.g. case-wise deletion, or use of secondary sources to impute missing data).		**N/A**
Information on the accuracy of the data e.g. via reference to one or more validation studies or acknowledgement that data accuracy is unknown.		**X**
**2. EXTRACTING FOOD OUTLETS**	**Essential**	**Desirable**
Description of methods used to extract food outlets of interest from dataset (e.g. search for specific proprietary classifications or store names).	**X**	
If outlets were extracted using search terms (e.g. proprietary classifications or store names):<br>• An exhaustive list of search terms (where proprietary classifications are used, it should be made explicitly clear that the classifications listed are those of the data provider).	**N/A**	
If outlets were extracted based on proprietary classifications:<br>• A copy of the proprietary classification scheme, optionally including exemplary outlets falling within each classification; OR,<br>• A discussion of any notable categories excluded from analyses (e.g. pubs, pharmacies, mobile food vendors etc.).		**X**
**3. DEFINING FOOD OUTLET CONSTRUCTS**	**Essential**	**Desirable**
Construct name(s) (e.g. 'supermarkets', 'healthy outlets', 'convenience stores' etc.).	**X**	
Description of the methods used to group outlets into constructs, including at least one of:<br>• An *exhaustive* list of any list-based criteria used to define each construct. This could include e.g. proprietary classifications making up each construct, or a list of store names making up each construct. Where proprietary classifications are used, it should be made explicitly clear that the classifications listed are those of the data provider.<br>• Any objective criteria e.g. floor space, number of tills etc. used to define constructs.<br>• Citation of any previously published categorisation schemes that have been applied to the data and description of the methods used to apply the scheme.<br>• Description of any other methods used (note methods based on subjective criteria are discouraged).	**X**	

Examples of outlets falling within each construct such that the scope of each construct can be more readily interpreted. For example, if the construct 'fast food outlet' includes 'traditional' burger and fried chicken outlets, and also coffee shops and sandwich shops then well-known chains falling within each such sub-type could be listed.	X	
Identification of any additional data sources used to group outlets into constructs e.g. use of Google Street View, business directories etc.		N/A
Description of how any additional data sources were linked to the food outlet data (e.g. by matching store names and/or addresses).		X
Where proprietary classifications are used to define constructs, a copy of the entire proprietary classification scheme.		N/A
**4. GEOCODING METHODS**	**Essential**	**Desirable**
Acknowledgement of whether any data has been geocoded.	X	
The address model used (e.g. areal unit, street segment, land parcel, address point).	N/A	
The match rate achieved.	N/A	
The environmental context, including details on how this was defined e.g. the study area was urban/rural, defined based on population density.	N/A	
Geocoding software used, including the version number.	N/A	
The source of geocoding reference data (e.g. street line segment data), including publication date.		N/A
**5. ACCESS METRICS**	**Essential**	**Desirable**
Definition of the conceptual environment being measured e.g. home, school, work etc.	X	
**Intensity Metrics**		
If areal zoning system used:<br>• The type of areal zoning system (e.g. government districts, census tracts etc.)<br>• The source of boundary data, including the publication date or other version identifier.	N/A	
If buffer zoning system used:<br>• The buffer size.<br>• The type of distance measure (e.g. Euclidian or network).	X	
The units of the intensity metric(s) (e.g. count per unit area, as measured in meters) or formula indicating how they were calculated.	X	
If network data was used (i.e. to calculate network distances):<br>• The source and publication date of network data.<br>• The types of road/path included.	N/A	
Rationale for the choice of zone type (e.g. areal vs buffer) and/or size as applicable.		X
**Proximity Metrics**		
The type of distance measure (Euclidian vs network).	N/A	
If network data was used (i.e. to calculate network distances):<br>• The source and publication date of network data.<br>• The types of road/path included.	N/A	
**Gravity Metrics**		
The zone radius.	N/A	
The decay coefficient.	N/A	
**6. UNKNOWN DETAILS**	**Essential**	**Desirable**
Any items noted as essential, but that are unknown should be highlighted as a limitation.	X	

To cite: Wilkins, E., Morris, M., Duncan, R. & Griffiths, C. (2016) Using Geographic Information Systems to measure retail food environments: discussion of methodological considerations and a proposed reporting checklist (Geo-FERN). **Health & Place**.
